# Comment on van Gemert et al. Asymptomatic Infant Rib Fractures Are Primarily Non-abuse-Related and Should Not Be Used to Assess Physical Child Abuse. *Children* 2023, *10*, 1827

**DOI:** 10.3390/children11101153

**Published:** 2024-09-24

**Authors:** Bernd Herrmann, Tanja Brüning, Sibylle Banaschak, Oliver Berthold

**Affiliations:** 1 Department for Neonatology and General Pediatrics, Klinikum Kassel GmbH, 34125 Kassel, Germany; herrmann@klinikum-kassel.de; 2 Department for Child Protection, Vestische Kinder- und Jugendklinik Datteln, Witten/Herdecke University, 45711 Datteln, Germany; t.bruening@kinderklinik-datteln.de; 3 Institute of Legal Medicine, University of Cologne, Faculty of Medicine and University Hospital, 50823 Cologne, Germany; sibylle.banaschak@uk-koeln.de; 4 Child Abuse Clinic, Department for Pediatrics, DRK Kliniken Berlin, 14050 Berlin, Germany

We are deeply concerned about the scientific quality of the article “Asymptomatic Infant Rib Fractures Are Primarily Non-abuse-Related and Should Not Be Used to Assess Physical Child Abuse” published in the journal *Children* in 2023 by van Gemert et al. [1].

From the Methods section, it is unclear what type of scientific work this article represents. The authors mentioned that they “scanned” publications in order to determine incidences of rib fractures due to non-abuse-related causes. As the authors do not refer to specific time periods, they are probably referring to prevalences rather than incidences [2]. This remains an issue throughout the whole manuscript. The authors mix alleged causes of childhood rib fractures with radiological findings that could allegedly be misdiagnosed as rib fractures. For conditions like “chronic placental histiocytic intervillositis”, not even a plausible correlation to rib fractures is presented, let alone a scientifically substantiated causality.

In this context, the authors commit a serious offence against scientific diligence and honesty. The correlation between “chronic placental histiocytic intervillositis” and rib fractures is claimed with reference to two articles (“Fragile bones likely develop, but rib fractures have been described rarely, we found 2 published cases [43,44]”) [3,4]. However, neither article mentions rib fractures. An innocent oversight seems unlikely here, as one of the co-authors of these articles [3] is also the co-author of this article [1].

The approach to references, which at best can be claimed to be confusing, is also demonstrated by the reference to the master’s thesis by Güvensel cited in the Introduction, which is not mentioned later on in the article [5]. However, the authors cite 1 of the supposedly 426 analyzed studies by Güvensel to make a point that child abuse research was “conducted with a flawed methodology”.

Various references are up to 60 years old (no. 32 from 1964, no. 26 and 34 from the 1980s; no. 18, 19 and 22 from the 1990s); due to the lack of a systematic search strategy, it is unclear why these articles were included. In view of the development of modern imaging techniques and the detection rate of infantile rib fractures up to 60 years later, it seems highly doubtful that they can contribute anything to clarify the questions raised [6,7,8,9,10,11] 

Apart from a reference to a narrative review by Gabaeff, also one of the co-authors of this article, the authors present no evidence of “rib cracks” [12].

In order to estimate “incidences” of rib fractures due to causes like “vitamin D deficiency”, “hypermobile Ehlers-Danlos syndrome” and “chronic placental histiocytic intervillositis”, the authors establish a hypothetical link between these diseases and osteogenesis imperfecta in order to deduce the “incidence” of rib fractures. This is just one example of how the authors work with so many detours that the connection between the question they ask and the answer they give can—from a scientific point of view—only be attributed to chance.

Another example is Section 3.1, where the authors try to determine the “incidence” of abusive rib fractures in children in the Netherlands by combining the “incidence” of child abuse in Belgium with the “incidence” of rib fractures due to resuscitation efforts in children from different countries.

As no systematic literature search was carried out, it remains unclear whether the selected references give representative data at all. Therefore, whether the combination of figures on child abuse in Belgium with figures on rib fractures after resuscitation efforts in children shows any correlation with the prevalence of abusive rib fractures in the Netherlands, as claimed by the authors, remains unanswered by scientific standards.

Contrary to the bold statement in the title, the authors make no distinction between symptomatic and asymptomatic rib fractures in the body of their manuscript, which is not surprising, as neither the cited references make such a distinction, nor is it clinically possible to differentiate reliably between symptomatic and asymptomatic rib fractures in preverbal children.

In addition, numerous claims are made that are not substantiated at all or only by “personal experience”. In this context, the claim that “the identification of such occult rib asymptomatic fractures in infants was frequently an undisputed sign that physical child abuse had taken place” is relevant in two respects. Firstly, it is wrong. In a meta-analysis by Maguire et al., rib fractures alone are associated with moderate predicted probability for the presence of abusive head trauma [13]. Secondly, it unmasks the real intent of the article. It is more of an opinion with a methodology that is not reproducible by other scientists and countless overly complex calculations that are based on unproven hypotheses and are intended to feign scientific accuracy.

In brief, this is an article that fails to produce a scientifically reproducible methodology, cannot logically substantiate the bold claim in the title and is filled with a multitude of calculations that are difficult to understand and whose relevance is not substantiated. We are severely concerned that this article is not written for medical professionals, but for courts in order to be able to question the relevance of rib fractures as a finding in child abuse cases. Therefore, this paper is likely to cause harm for the safeguarding of children who have been abused, and it is already causing significant harm to the scientific reputation of *Children* (Basel) and the respected scholars on the Editorial Board. For the reasons stated above and to avoid further harm, we recommend that you consider a retraction of this article.
